# Lactulose-Induced Transcriptional Reprogramming and Repression of Persistence-Associated Phenotypes in Non-Typhoidal *Salmonella enterica*

**DOI:** 10.3390/pathogens15060592

**Published:** 2026-06-01

**Authors:** Juned Ahmed, Samuel Ajulo, Smriti Shringi, Bradd J. Haley, Devendra H. Shah

**Affiliations:** 1 School of Veterinary Medicine, Texas Tech University, Amarillo, TX 79106, USAsmriti.shringi@ttu.edu (S.S.); 2 Environmental Microbial and Food Safety Laboratory, Beltsville Agricultural Research Center, Agricultural Research Service, United States Department of Agriculture, Beltsville, MD 20705, USA

**Keywords:** *Salmonella*, lactulose, metabolism, transcriptomics, stress, biofilm, motility

## Abstract

Lactulose, a synthetic disaccharide widely used in human and veterinary medicine as a therapeutic and prebiotic, is generally assumed to be metabolized exclusively by commensal microbiota. We recently showed that non-typhoidal *Salmonella* (NTS) can also metabolize lactulose, but the genetic basis and functional consequences remain unclear. Here, we hypothesized that NTS encodes specific genetic determinants and regulatory networks for lactulose uptake, hydrolysis, and downstream metabolism. Using the globally disseminated NTS serotype *S.* Kentucky, we defined the lactulose-responsive transcriptome. Lactulose exposure induced a distinct gene network including candidate transporters, periplasmic binding proteins, and glycoside hydrolases predicted to cleave lactulose into galactose and fructose, with downstream metabolism via the mannitol cycle and Leloir-associated pathways. Notably, lactulose also triggered broad transcriptional remodeling that repressed genes encoding motility, fimbrial biosynthesis, and biofilm formation, resulting in significantly reduced motility and biofilm across NTS serotypes. These findings provide novel mechanistic insight into lactulose metabolism, suggesting that lactulose metabolism is concurrently associated with reduced expression of persistence-associated phenotypes in NTS under the conditions tested. This raises the possibility that lactulose exposure could influence NTS persistence in both the host-associated and environmental context. Follow-up studies are needed to validate the potential of these findings to reduce food safety and public health risks linked to persistence and transmission phenotypes.

## 1. Introduction

Lactulose, a synthetic disaccharide composed of fructose and galactose moieties linked by a β 1,4 glycosidic bond, is widely used in human and veterinary medicine as a prebiotic and for the therapeutic management of hepatic encephalopathy, constipation, and intestinal dysbiosis [1,2,3,4,5,6]. Lactulose is also used as a sugar substitute in the dairy, bakery, confectionery, and pharmaceutical industries [7]. The prebiotic potential of lactulose in improving gut health and reducing gastrointestinal colonization by potential pathogens has been tangentially investigated in humans, rodents, and food animals, such as swine and poultry [8,9,10,11,12]. However, its efficacy in controlling enteric pathogens is less well established, and published data show that its impact on enteric pathogens remains inconsistent across different host and bacterial species [10,11,12,13,14,15,16]. Lactulose is believed to be primarily fermented by commensal gut microbiota and improve gut health via acidification of the colonic environment, production of volatile fatty acids, and alterations in nitrogen and osmotic balance [17,18,19,20,21]. However, the accumulating evidence reveals that strains of opportunistic pathogens, such as *Enterococcus*, *Streptococcus*, *Escherichia*, *Pseudomonas*, *Klebsiella*, and *Cronobacter,* can also metabolize lactulose [22], suggesting that lactulose metabolism is not restricted to commensals. Recently, we reported that enteric pathogens such as non-typhoidal *Salmonella* (NTS) can also metabolize lactulose in a serotype-independent manner [23], raising the possibility that NTS may carry the genetic framework needed for lactulose metabolism; however, the underlying genetic basis remains unclear.

This study was designed to determine the genetic basis of lactulose metabolism in NTS. We hypothesized that NTS harbors genetic determinants and a regulatory network required for lactulose uptake, cleavage, and downstream routing for energy production. We tested this hypothesis by comparative profiling of the lactulose-induced transcriptome of the globally emerging NTS serotype *Salmonella* Kentucky (*S.* Kentucky), a relevant and tractable model pathogen previously known to metabolize lactulose as an energy source [23,24]. In this study, we show that lactulose exposure induces extensive transcriptional reprogramming in NTS, with two concurrent, mechanistically linked outcomes. First, lactulose triggers metabolic reprogramming in NTS that reveals a functional pathway for its uptake, cleavage, and flexible downstream routing of constituent sugars into central metabolism, demonstrating that NTS can metabolize lactulose as an energy source. Concurrently, lactulose metabolism also induces repression of persistence-associated genetic networks encoding curli and cellulose-mediated biofilm formation, flagella-mediated motility, and type-1 fimbrial operons involved in attachment and intestinal colonization. The lactulose-induced repression of genetic networks associated with biofilm and motility is directly translated into suppression of the corresponding phenotypes in NTS independent of serotypes. Collectively, this study provides insights into lactulose metabolism in NTS and how lactulose induces genetic reprogramming of persistence-associated phenotypes of NTS, with potential implications for pathogen ecology in host and environmental contexts.

## 2. Materials and Methods

### 2.1. Strain Selection and Growth Conditions for RNA-Seq

Seven epidemiologically distinct strains of *S.* Kentucky (ST152, *n* = 4; ST198, *n* = 3) were used for transcriptomic profiling (Table 1). Frozen stocks (−80 °C) were streaked on tryptic soy agar (TSA) and incubated at 37 °C for 16 h. Colonies (~10^9^ CFU) were inoculated into M9 (1×) broth (BD Difco, Franklin Lakes, NJ, USA) (Na_2_HPO_4_, KH_2_PO_4_, NaCl, NH_4_Cl; supplemented with 2 mM MgSO_4_ and 0.1 mM CaCl_2_) containing 20 mM lactulose (ThermoFisher Scientific, Waltham, MA, USA, Cat #J60160.18) or 20 mM glucose (control) and were subjected to short-term incubation (6 h) at 37 °C with shaking (200 rpm). Cell densities were normalized to ~10^9^ CFU, pelleted (10,000× *g*, 4 °C, 10 min), washed with sterile PBS (1×), and processed immediately for RNA extraction or stored at −80 °C in RNAlater™ (Invitrogen by ThermoFisher Scientific, USA). Upon thawing, pellets were diluted 1:1 with PBS (1×), centrifuged (10,000× *g*, 4 °C, 10 min), and washed once with PBS (1×) to remove residual preservative before the extraction of total RNA.

### 2.2. RNA Isolation, Cleanup, Library Preparation, and Sequencing

Cell pellets were lysed in 1 mL TRI Reagent (Zymo Research, Irvine, CA, USA) and incubated at room temperature for 5 min. Total RNA was purified using the Direct-zol™ RNA Miniprep Plus kit (Zymo Research, USA) per manufacturer’s protocol with minor modifications. On-column DNase digestion was performed with TURBO™ DNase (Invitrogen by ThermoFisher Scientific, USA) (8–10 U, 37 °C, 30 min), followed by cleanup using the RNA Clean & Concentrator™-25 kit (Zymo Research, USA). RNA integrity and concentration were assessed with an Agilent 4200 TapeStation (Agilent Technologies, Inc., Santa Clara, CA, USA). Genomic DNA carryover was evaluated by PCR targeting *invA* using primers and conditions as described previously [26], with products resolved on 1.5% agarose gels. Sequencing libraries (Novogene Corporation Inc., Sacramento, CA, USA) were prepared after removal of <200-nt RNA fragments (RNAClean XP; Beckman Coulter, Brea, CA, USA) and rRNA depletion (QIAseq FastSelect 5S/16S/23S; Qiagen, Germantown, MD, USA), followed by strand-specific library construction (NEBNext Ultra II Directional RNA Library Prep; New England Biolabs, Ipswich, MA, USA). Libraries (~200-bp inserts) were sequenced on an Illumina NovaSeq X Plus 10B platform (Illumina, Inc., San Diego, CA, USA).

### 2.3. RNA-Seq Data Processing and Differential Expression Analysis

Raw paired-end (PE) reads were adapter and quality trimmed using fastp [27]; quality metrics were assessed using FastQC (v0.11.9) [28]. Filtered reads were aligned to the *S.* Kentucky PU131 reference genome (NCBI RefSeq: https://www.ncbi.nlm.nih.gov/nuccore/NZ_CP026327.1 (accessed on 9 November 2025)) using Bowtie2 (v2.5.x) [29]. SAM files were converted to sorted, indexed BAM files with SAMtools (v1.17) [30]. Gene-level counts were generated using featureCounts (version 2.1.0) [31] and imported into RStudio (version 4.4.1) for differential expression (DE) analysis with edgeR [32]. Significance thresholds included log_2_FC ≥ 1, logCPM ≥ 3.5, *p* < 0.05, and FDR < 0.05 for lactulose vs. glucose. Of 4769 total genes, 4499 protein-coding genes were retained for DE analysis.

### 2.4. Functional Annotation and Enrichment of RNA-Seq Data

GenBank annotations (GBFF) for *S.* Kentucky PU131 reference genome (https://www.ncbi.nlm.nih.gov/nuccore/NZ_CP026327.1 (accessed on 9 November 2025)) were parsed to extract locus tags, gene symbols, product descriptions, and amino acid sequences using Biopython. The locus tags were then mapped to the DE results to integrate functional metadata. Amino acid sequences encoded by DE genes were analyzed with eggNOG-mapper v2 to assign COG functional categories [33,34], and protein–protein associations were explored using STRING [35]. These analyses informed pathway-level interpretations and network-level context for the DE genes encoding putative lactulose transporter and hydrolase candidates.

### 2.5. Subcellular Localization and Structural Predictions of Proteins of DE Genes

Subcellular localization of putative transporters was predicted using PSORTb v3.0.3 and SignalP 6.0 [36,37]. Representative outer-membrane porins were modeled using AlphaFold (AlphaFold Server), and the model confidence was evaluated by pLDDT [38,39]. Structural renderings were used qualitatively to illustrate β-barrel architecture and support transporter candidacy.

### 2.6. Biofilm Assays

#### 2.6.1. Congo Red–Coomassie Brilliant Blue (CRCBB) Assay

The CRCBB assay provides a qualitative readout of curli plus cellulose-dependent biofilm matrix, where curli-producing NTS typically exhibits *rdar* (rough, dry, and red) colony morphology [25,40,41]. Six well-characterized biofilm-positive NTS strains that typically exhibit *rdar* colony morphology were streaked on LB agar and incubated at 37 °C for 24 h (Table 1). A single colony per strain was inoculated into LB broth and grown overnight (37 °C, 200 rpm). Aliquots (5 µL) were spotted (in triplicate) onto LB no-salt (LBNS) agar supplemented with lactulose (20 mM), glucose (20 mM), or no supplement (control). All plates contained Congo red (40 µg/mL) and Coomassie brilliant blue (20 µg/mL). Plates were incubated at 25 °C for 96 h. Colony macro-morphology (color/roughness) was recorded visually; microscopic features were imaged at 4× using a light microscope (Omax Microscopes, Irvine, CA, USA), as described previously [25].

#### 2.6.2. Calcofluor Assay

The Calcofluor assay specifically provides a readout for cellulose-dependent biofilm matrix, where cellulose-producing NTS exhibits strong fluorescence upon exposure to long-wave UV light [25,42]. Briefly, overnight cultures prepared as above were spotted (5 µL; triplicate) onto LBNS agar containing Calcofluor (200 µg/mL) with lactulose (20 mM), glucose (20 mM), or no supplement and incubated at 25 °C for 72 h. Fluorescence was scored after exposure to long-wave UV (366 nm) as fluorescence intensity on a 0 to 3 ordinal scale by four observers blinded to the treatment groups. Scores were analyzed by pairwise Wilcoxon rank-sum tests with Holm adjustment for multiple comparisons. In some strains, replicate scores exhibited zero variance (e.g., 2, 2, 2 or 1, 1, 1), violating parametric assumptions; therefore, non-parametric tests were applied to ensure valid inference.

### 2.7. Motility Assay

To determine the impact of lactulose on the flagella-dependent motility of NTS, motility assays were performed by measuring movement on semi-solid agar and microscopic evaluation of bacterial movement in liquid cultures. Briefly, seven representative strains of *S.* Kentucky (Table 1) were revived by streaking on TSA and incubated at 37 °C for 16 h. After overnight incubation, colonies were resuspended in M9 (1×) broth supplemented with 20 mM of lactulose or glucose (control) and incubated for 6 h at 37 °C with 200 rpm shaking. Next, the OD of each culture was measured after 6 h incubation using a densitometer (DEN-1B, Grant Bio, Beaver Falls, PA, USA) and normalized.

For microscopic evaluation, 8 µL of normalized cultures were loaded onto a counting chamber (Fisher Scientific, Pittsburg, PA, USA) and imaged at 40× using a compound light microscope (Omax, USA). The videos (.MOV) were captured at 1080p resolution using a 13 mm f/2.2 lens at 29.98 Frames Per Second (fps), cropped to a consistent field, and quantitatively analyzed using YSMR for the 2D tracking of individual cells [43]. For each strain and nutrient condition, three biological replicates were recorded. Total travel distance and instantaneous speed were extracted; per cell metrics comprised 841 individual cells in the glucose-supplemented condition and 1134 individual cells in the lactulose-supplemented condition, and data were analyzed by paired, within-strain comparisons using the Wilcoxon signed-rank test. The motility assays were expanded to other NTS serovars (non-Kentucky) to determine the serovar-level effects.

Soft agar swimming motility was assessed on 0.3% agar plates prepared with M9 (1×) supplemented with lactulose (20 mM) or glucose (20 mM), following procedures described previously with minor modifications [44,45,46]. Briefly, cultures (1.5 µL; ~2 × 10^6^ CFU) from the 6 h incubations were stab-inoculated into the agar and incubated at 37 °C for 20 h. Migration was quantified as the diameter of the motility zone (mm). Group means were compared using a Student’s *t*-test in RStudio (version 4.4.1) using a *p*-value < 0.01 as statistically significant.

## 3. Results and Discussion

### 3.1. Lactulose Induces Extensive Transcriptional Reprogramming in Salmonella

**Figure 1:**
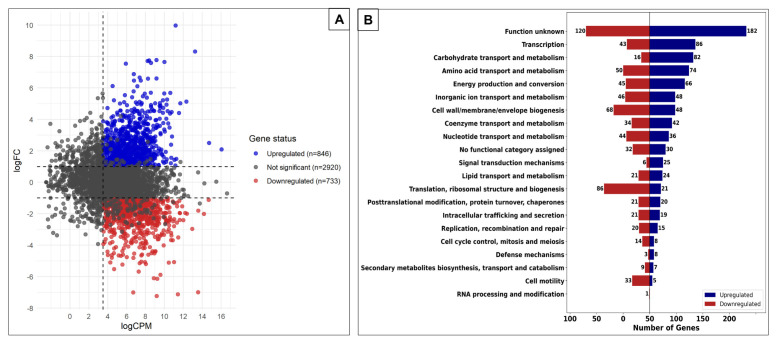
(**A**) The mean average (MA) plot showing the distribution of the differentially expressed genes in NTS exposed to lactulose when compared with glucose. The blue dots represent upregulated genes, red dots represent downregulated genes, and grey dots represent genes that are not significantly differentially expressed. The significance thresholds for differential expression (logFC = ±1 and logCPM = 3.5) are indicated with dashed lines. (**B**) Clusters of orthologous groups (COG) and functional classification of upregulated (blue) and downregulated (red) genes derived using eggNOG-mapper v2. The numbers on each bar represent the total number of genes within each COG category.

Comparative profiling of the lactulose-induced transcriptome with the glucose-induced transcriptome of NTS revealed that lactulose exposure triggers widespread transcriptional reprogramming. Out of 4499 protein-coding genes, the expression of 866 genes was significantly upregulated, whereas 733 genes were downregulated (Figure 1A and Supplementary File S1), indicating that lactulose elicits a broad transcriptional reprogramming. Functional enrichment analysis showed that genes involved in carbohydrate transport and metabolism were disproportionately represented among upregulated genes, consistent with bacterial adaptation to lactulose as a non-preferred but utilizable synthetic carbon source (Supplementary File S2). In contrast, genes associated with cell motility, cell envelope biogenesis (including biofilm and fimbrial biosynthesis), ribosomal structure, biogenesis, and translational machinery were disproportionately represented among downregulated categories (Figure 1B). Collectively, this pattern (Figure 1B) is consistent with a trade-off between energy scavenging via lactulose metabolism and lactulose exposure mediating changes in the expression of persistence-associated genetic networks. Because glucose is a preferred carbon source with known effects on global regulatory pathways, some observed differences may reflect glucose-driven regulatory states in addition to lactulose-specific responses.

### 3.2. Lactulose Catabolic Pathway in NTS

**Figure 2:**
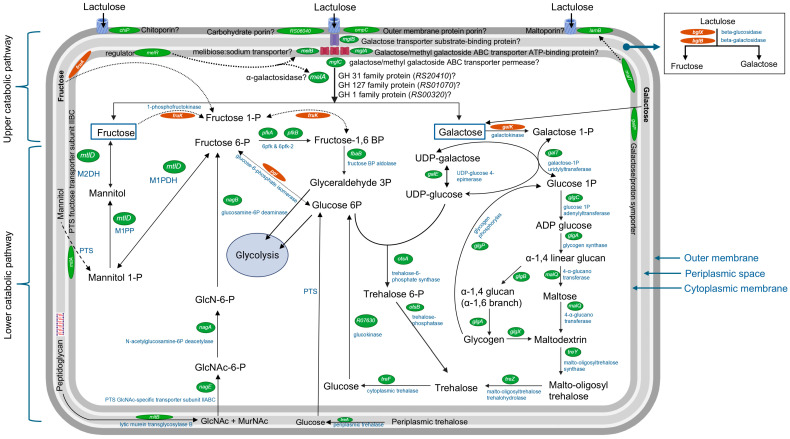
The proposed metabolic pathway of lactulose metabolismin NTS was constructed based on differential gene expression analysis, functional prediction, and cellular localization. Genes upregulated in response to lactulose are circled in green, and their product/enzyme names are shown as blue text. Genes showing higher logCPM expression in the presence of lactulose are highlighted with orange circles; however, these genes do not meet all the defined thresholds for differential expression (logFC = ±1 and logCPM = 3.5). The figure was created with Biorender (biorender.com).

**Figure 3:**
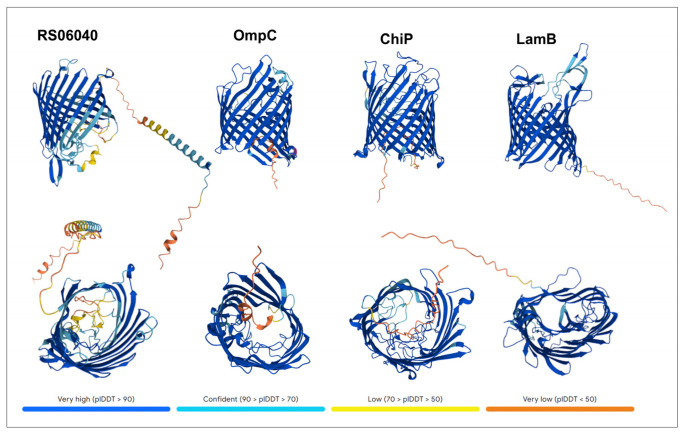
AlphaFold-predicted 3D structures of outer membrane proteins (RS06040, OmpC, ChiP, and LamB) showing conserved β-barrel architecture and overall fold topology. Structures are shown in the side view (top) and top–down view (bottom). Models were generated using the AlphaFold web server and are colored by predicted local distance confidence test (pLDDT).

Based on functional analysis of the lactulose exposure-induced transcriptome, we constructed a proposed transcriptome-guided putative lactulose catabolic pathway that integrates the predicted uptake, cleavage, and downstream metabolic routing of lactulose in NTS (Figure 2). The proposed pathway shows that lactulose uptake is likely mediated through promiscuous outer-membrane porins and galactoside-oriented transport systems rather than a dedicated lactulose transporter (Figure 3). Following uptake, the β-1,4 glycosidic bond of lactulose is likely cleaved by multiple putative glycoside hydrolases in the periplasm and cytoplasm, releasing galactose and fructose. Downstream metabolism of galactose and fructose then proceeds through non-canonical fructose metabolism via the mannitol cycle, together with multidirectional galactose flux through the Leloir and trehalose biosynthesis pathways, underscoring the metabolic plasticity of NTS during lactulose metabolism.

#### 3.2.1. Lactulose Uptake via Promiscuous Transport Systems in NTS

Transcriptional profiling revealed that lactulose induces a coordinated remodeling of several genes encoding carbohydrate-permissive β-barrel porins, including *ompC*, *lamB*, *chiP*, and *RS06040* (Figure 2 and Supplementary File S1). AlphaFold structural predictions of RS06040, OmpC, ChiP, and LamB revealed conserved β-barrel architectures characteristic of carbohydrate-permeable outer-membrane porins (Figure 3). Unlike α-helical transmembrane proteins, which rely on continuous hydrophobic stretches to span the lipid bilayer, β-barrel proteins feature alternating hydrophobic and hydrophilic residues that create a cylinder with a hydrophobic exterior anchored in the membrane and a hydrophilic interior forming an aqueous transmembrane channel, a structural arrangement compatible with nutrient transport across membranes, consistent with potential roles of RS06040, OmpC, ChiP, and LamB in lactulose influx [47,48].

Interestingly, lactulose-induced upregulation of *ompC* was coupled with downregulation of *ompF* (Supplementary File S1), indicating that NTS is likely exhibiting an osmoadaptive response classically seen under elevated osmolarity or ionic strength. This shift likely arises because glucose is rapidly metabolized, whereas lactulose is degraded more slowly, leaving higher residual sugar concentrations that may increase osmotic pressure, thereby increasing permeability of OmpC to aid sustained uptake of larger solutes such as lactulose [49,50]. In contrast, although the gene encoding the OmpL was upregulated, OmpL porin does not permit uptake of the similarly sized disaccharide maltose due to pore size constraints [51] and is therefore unlikely to admit lactulose, which has a molecular weight similar to maltose. Collectively, these data support an OmpF-to-OmpC switch consistent with altered envelope and osmotic conditions in lactulose-supplemented media, which collectively facilitate the initial stages of lactulose uptake (Figure 2).

Besides this osmoadaptive *ompF-ompC* porin switch, genes encoding several other carbohydrate-permissive β-barrel porins were induced, including *RS06040*, *lamB*, and *chiP* (Figure 2). The *RS06040* gene encodes a porin homologous to RafY from *Escherichia coli* (58% identity and 99% coverage), a protein known to mediate uptake of raffinose family oligosaccharides [52]. Given its predicted substrate profile and strong upregulation, RS06040 likely represents a strong candidate for facilitating lactulose entry into the periplasm. Lactulose-induced expression of *chiP* (Figure 2) coupled with the *chbCARG* genes (Supplementary File S1), which are classically associated with transport and degradation of β-linked chitooligosaccharides [53,54,55]. This upregulated expression of *chiP* in the absence of chitosugars suggests that ChiP porin may recognize lactulose as a structurally related substrate, thereby contributing to its uptake. While this interpretation is consistent with the substrate promiscuity observed for several carbohydrate transport systems, targeted transport assays will be required to confirm a direct role for ChiP in lactulose uptake. Finally, the lactulose-induced upregulation of *lamB* is similarly noteworthy. LamB is best known for transporting maltodextrins; it also permits the influx of a broad range of carbohydrates, including disaccharides [56]. Expression of *lamB* is controlled by the central regulator *malT* [57,58], which was also significantly upregulated in response to lactulose (Figure 2 and Supplementary File S1). This coordinated *lamB-malT* induction suggests that lactulose also engages maltose-responsive regulatory networks, further supporting the notion that NTS exploits existing carbohydrate uptake systems rather than a dedicated lactulose-specific transporter.

Post-uptake, a few upregulated genes, such as *bglX* and *blgB* (Figure 2)*,* encoding periplasmic membrane proteins likely facilitate periplasmic cleavage and transport into the cytoplasm. Periplasmic β-glucosidase BglX exhibits broad cleavage activity against different galactose-containing substrates, including lactose, which has a similar molecular weight to lactulose and a galactose moiety linked with a glycosidic bond [59]. Similarly, *bglB*, which encodes β-galactosidase, is known to hydrolyze β-1,4-glycosidic bonds in the periplasm [60]. While the logFC of these two genes was not statistically significant, higher logCPM and number of corresponding transcripts indicate that the genes are abundantly transcribed both in lactulose and glucose (control), suggesting that cleavage of lactulose may occur in the periplasm, a hypothesis that may warrant follow-up biochemical functional investigations.

Lactulose exposure upregulated the periplasmic substrate-binding protein gene *mglB* (Figure 2), which is classically associated with high-affinity galactose and glucose transport [61]. This likely reflects recognition of free galactose or the galactose moiety of lactulose. In parallel, three genes of the *mel* operon (*melB*, *melA*, and *melR*), which mediate melibiose transport and catabolism, were significantly induced (Figure 2). Transcription of the *mel* operon is activated by MelR and cyclic AMP receptor protein (CRP), which bind adjacent sites in the *melAB* promoter encoding the α-galactoside symporter MelB and α-galactosidase MelA [62,63,64,65]. Notably, MelB exhibits broad substrate specificity for both α- and β-galactose anomers [66], and *mel* operon induction has been reported in response to β-galactosides such as lactose [64]. Together, the strong induction of *melB* and the high-affinity galactose transporter MglABC support enhanced scavenging of galactosides or galactose by NTS. This reliance on substrate-promiscuous transport systems is consistent with metabolic adaptation to structurally related sugars in competitive environments [67]. However, targeted mutagenesis and transport assays will be required to determine their direct roles in lactulose uptake. Finally, lactulose altered the expression of over 100 additional transporters (e.g., *manXYZ*, *nagE*, *srlAE*, *mtlA*) involved in the uptake of carbohydrates, amino acids, and minerals (Supplementary File S1), indicating a broader carbon-scavenging and stress-responsive state rather than specialization for lactulose alone.

#### 3.2.2. Transcriptomic Evidence for Lactulose Cleavage by Multiple Glycoside Hydrolases

Lactulose is a synthetic disaccharide of fructose and galactose linked by a β-1,4 glycosidic bond [5]. Its utilization requires hydrolysis of this bond to release monosaccharides for central metabolism. This step is catalyzed by glycoside hydrolases (glycosidases) and galactosidases that cleave β-1,4 linkages [68,69,70]. Transcriptomic analysis revealed that lactulose induced differential expression of at least 33 genes encoding hydrolase enzymes with known, predicted, or unknown substrate specificity (Supplementary File S1). Among these, four genes were significantly upregulated and are likely to play major roles in the cytoplasmic cleavage of lactulose. These included *melA*, encoding an α-galactosidase; *RS20410*, encoding a glycoside hydrolase family 31 (GH31) protein; *RS01070*, encoding a GH127 family protein; and *RS00320*, encoding a GH1 family protein (Figure 2). Although *melA* is classically annotated as an α-galactosidase involved in melibiose catabolism, it was among the most highly upregulated genes under lactulose supplementation (logFC = 9.97; logCPM = 11.2) (Supplementary File S1). Importantly, *melA* expression is also regulated in response to lactose, a structurally related β-galactoside linked by a β-1,4-glycosidic bond [64]. Three other upregulated genes encoding putative glycoside hydrolase GH31, GH127, and GH1 family proteins are also likely to contribute to lactulose cleavage in the cytoplasm. Although the precise substrate specificities of these proteins remain uncharacterized, the concurrent upregulation of multiple glycoside hydrolases suggests a redundant cleavage strategy, which may enhance metabolic robustness and allow NTS to process lactulose under varying environmental conditions efficiently.

Collectively, these data indicate that lactulose cleavage in NTS is unlikely to be mediated by a single specialized enzyme but instead relies on overlapping activities of multiple glycoside hydrolases. This redundancy is consistent with the broader metabolic plasticity observed during lactulose utilization and supports the pathway model summarized in Figure 2, in which uptake, cleavage, and downstream routing are integrated into existing carbohydrate metabolic networks. Targeted mutagenesis, transport assays, and biochemical validation will be necessary to define the roles of the putative hydrolases identified in this study.

#### 3.2.3. Non-Canonical Routing of Lactulose-Derived Sugars into Central Metabolism

Lactulose-induced expression of several genes involved in the downstream catabolism of fructose and galactose (Figure 2). Canonically, fructose is phosphorylated to fructose-1-phosphate by 1-phosphofructokinase (FruK); however, FruK can also act on fructose-1,6-bisphosphate to generate fructose-1-phosphate [71,72,73,74]. Periplasmic fructose is transported into the cytoplasm via FruA, a PTS fructose transporter subunit IIBC [75]. Although *fruK* and *fruA* were not differentially expressed under lactulose supplementation despite high basal expression (Supplementary File S1), this pattern suggests fructose derived from lactulose hydrolysis is primarily routed through a non-canonical pathway. Consistently, genes of the mannitol cycle were transcriptionally activated, supporting an alternative route for fructose assimilation. In this pathway, fructose is reversibly reduced to mannitol via mannitol 2-dehydrogenase (MtlD), while fructose-6-phosphate is interconverted with mannitol-1-phosphate through MtlD-associated activities, ultimately regenerating fructose-6-phosphate [76,77]. Fructose-6-phosphate then enters glycolysis via conversion to fructose-1,6-bisphosphate by phosphofructokinase, followed by cleavage to glyceraldehyde-3-phosphate by fructose-bisphosphate aldolase (FbaB) [78,79]. Collectively, these data support preferential routing of lactulose-derived fructose through a mannitol-associated cycle before central carbon metabolism (Figure 2).

Galactose, the second monosaccharide released from lactulose hydrolysis, is primarily metabolized via the Leloir pathway (Figure 2). Consistent with this, the *galP* gene encoding GalP, a proton symporter responsible for periplasmic galactose uptake, was significantly upregulated (Figure 2 and Supplementary File S1) [80]. Intracellularly, galactose is phosphorylated by GalK to galactose-1-phosphate, which is then converted to UDP-galactose or glucose-1-phosphate by GalT (Figure 2). UDP-galactose is subsequently epimerized to UDP-glucose by GalE [81,82,83].

Downstream of the Leloir pathway, galactose metabolism diverges into multiple branches rather than a single linear route. UDP-glucose can be converted to trehalose-6-phosphate via OtsA and subsequently dephosphorylated to trehalose by OtsB [83]. In parallel, glucose-1-phosphate is converted to ADP-glucose by GlgC, feeding glycogen biosynthesis via GlgA, GlgB, and GlgX [84,85]. Maltodextrins can also be generated through MalQ-mediated reactions and further processed via the TreY/TreZ pathway to trehalose, which is ultimately hydrolyzed by TreF to glucose [86,87]. Glucose is then phosphorylated by glucokinase to glucose-6-phosphate, enabling entry into glycolysis or re-entry into storage pathways [83]. Together, these data indicate that lactulose-derived galactose is distributed through a highly branched network linking the Leloir pathway to trehalose metabolism, glycogen synthesis, and central carbon metabolism (Figure 2).

In addition, lactulose significantly induced expression of *uxaC*, *uxuB*, *uxuA*, and *uxuR*, indicating activation of the hexuronate pathway (Supplementary File S1) [88]. Galactose metabolism generates multiple intermediates, including glucose-1-phosphate, glucose-6-phosphate, ADP-glucose, and free glucose (Figure 2), which can feed into D-glucuronic acid biosynthesis via the hexuronate pathway [89]. Although *ugd*, encoding UDP-glucose dehydrogenase, was significantly downregulated (−3.4 fold) under lactulose exposure (Supplementary File S1) [90,91,92], the induction of other hexuronate genes suggests compensatory enzymatic routes or alternative regulatory control.

#### 3.2.4. Lactulose-Induced Reprogramming of Peptidoglycan Recycling and Amino Sugar Metabolism

Lactulose-induced expression of genes such as *mtlB* and *nagEAB* intersects with cell wall sugar recycling pathways (Figure 2). The bacterial peptidoglycan layer contains N-acetylglucosamine (GlcNAc) and N-acetylmuramic acid (MurNAc) linked by a β-1,4 glycosidic bond. Lactulose-induced overexpression of the *mltB* gene encodes lytic murein transglycosylase B, which cleaves this linkage and releases muropeptide-derived GlcNAc-containing fragments that are subsequently processed through the cell wall recycling pathway. Free GlcNAc is transported via the phosphotransferase system (PTS) GlcNAc-specific transporter encoded by *nagE*, resulting in concomitant phosphorylation to GlcNAc-6-P (Figure 2). GlcNAc-6-P is then deacetylated by NagA (N-acetylglucosamine-6-phosphate deacetylase) to glucosamine-6-phosphate, which represents a central branch point between anabolic recycling and catabolic metabolism. Under canonical conditions, glucosamine-6-phosphate is channeled into peptidoglycan precursor biosynthesis via GlmMU to generate UDP-GlcNAc, which subsequently feeds into UDP-MurNAc formation through MurAB and downstream assembly steps catalyzed by MurCDEFGJ [93]. However, in NTS exposed to lactulose, several genes within the *glm* and *mur* pathways are repressed (Supplemental File S1), potentially limiting flux from recycled amino sugars into UDP-GlcNAc and downstream peptidoglycan biosynthesis. Concomitantly, lactulose-induced upregulation of *nagB* encoding glucosamine-6-phosphate deaminase can be expected to divert glucosamine-6-phosphate away from cell wall precursor synthesis by converting it into fructose-6-phosphate [94], thereby funneling recycled amino sugars into central carbon metabolism (Figure 2). Collectively, these transcriptional patterns indicate that amino sugar recycling is redirected away from peptidoglycan biosynthesis toward glycolysis-linked metabolism during lactulose utilization, underscoring the integration of cell wall turnover with broader carbohydrate metabolism and central carbon flux in NTS.

### 3.3. Lactulose-Induced Genotypic and Phenotypic Repression of Curli-Dependent Biofilm Phenotype

**Figure 4:**
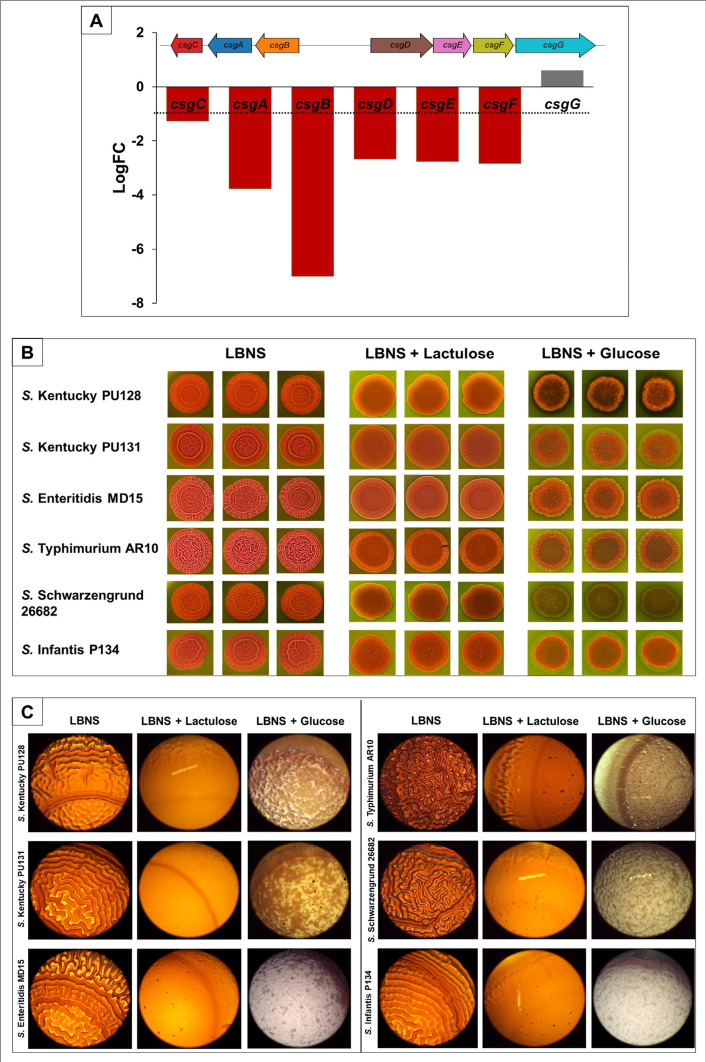
Lactulose exposure represses expression of the *csg* operon and suppresses curli-associated colony morphology in non-typhoidal *Salmonella* (NTS). (**A**) Expression of the *csg* operon, which regulates curli biosynthesis, is reduced in *S.* Kentucky following lactulose exposure. Red bars indicate genes that are significantly downregulated based on differential expression criteria (log_2_FC ≥ 1/≤−1, logCPM ≥ 3.5, and adjusted *p* value/FDR threshold), whereas the gray bar denotes genes that are not differentially expressed. The dotted line indicates the log_2_ fold-change threshold (log_2_FC = 1). (**B**) Macroscopic colony morphology on CRCBB agar demonstrates that supplementation with 20 mM lactulose (LBNS + lactulose) suppresses the *rdar* (rough, dry, and red) morphotype across all tested NTS serotypes. The *rdar* phenotype, indicative of curli production in biofilm-forming strains, is reduced in lactulose-supplemented media (LBNS + lactulose), resulting in smoother colonies compared to controls (LBNS or LBNS + glucose). (**C**) Microscopic analysis (4× objective) of colonies on CRCBB agar shows a pronounced *rdar* morphotype in NTS serotypes grown in LBNS or LBNS + glucose. In contrast, colonies grown in LBNS + lactulose exhibit smoother surfaces, reduced wrinkling, and occasional shallow crater-like features, consistent with decreased curli production.

Lactulose induced the differential expression of at least 60 biofilm-associated genes, of which 22 were upregulated, 14 were downregulated, and 24 showed no significant change (Supplementary File S1). Among biofilm-associated genes, curli fimbriae, encoded by the *csg* operon and regulated by *csgD* [95], form key structural components of the *Salmonella* extracellular matrix that mediate surface attachment, aggregation, and biofilm architecture [96,97,98]. CsgD-regulated curli production contributes to the characteristic red, dry, and rough (*rdar*) morphotype on Congo red–Coomassie brilliant blue (CRCBB) agar [25,99]. In this study, lactulose exposure resulted in consistent downregulation of the expression of the entire *csgCABDEF* operon (Figure 4A). Importantly, the transcriptomic analysis was conducted at mammalian physiological temperature (37 °C), which is not inherently considered a biofilm-inducing condition. Therefore, the repression of biofilm-associated *csg* genes observed here is likely attributable to lactulose-induced transcriptional reprogramming, rather than secondary effects of biofilm-permissive growth conditions. The lactulose-induced downregulation of the *csg* operon was accompanied by suppression of the *rdar* morphotype on CRCBB agar (Figure 4B). This phenotypic effect was consistent across all tested NTS serotypes, with lactulose-treated colonies displaying smoother morphology and reduced Congo red binding (Figure 4B,C). In contrast, glucose-supplemented colonies retained *rdar* morphology, albeit with color changes associated with medium acidification [100,101]. The observed repression of curli is consistent with prior studies demonstrating that amino sugar metabolism, particularly elevated GlcNAc-6-phosphate, can modulate curli expression in *Escherichia coli* by repressing *csgBA* and *csgDEFG* transcription and reducing curli production [102]. In the present study, lactulose-induced transcriptional changes, including repression of peptidoglycan biosynthetic (*glm/mur*) pathways (Supplemental File S1) and upregulation of *nagB* (Figure 2), suggest potential metabolic rerouting of glucosamine-6-phosphate toward central carbon metabolism rather than cell envelope precursor synthesis. This shift in amino sugar utilization is therefore consistent with a metabolic state previously associated with reduced curli expression.

### 3.4. Lactulose Induced the Expression of Genes Encoding Cellulose, Coupled with a Net Reduction in Cellulose Production

**Figure 5:**
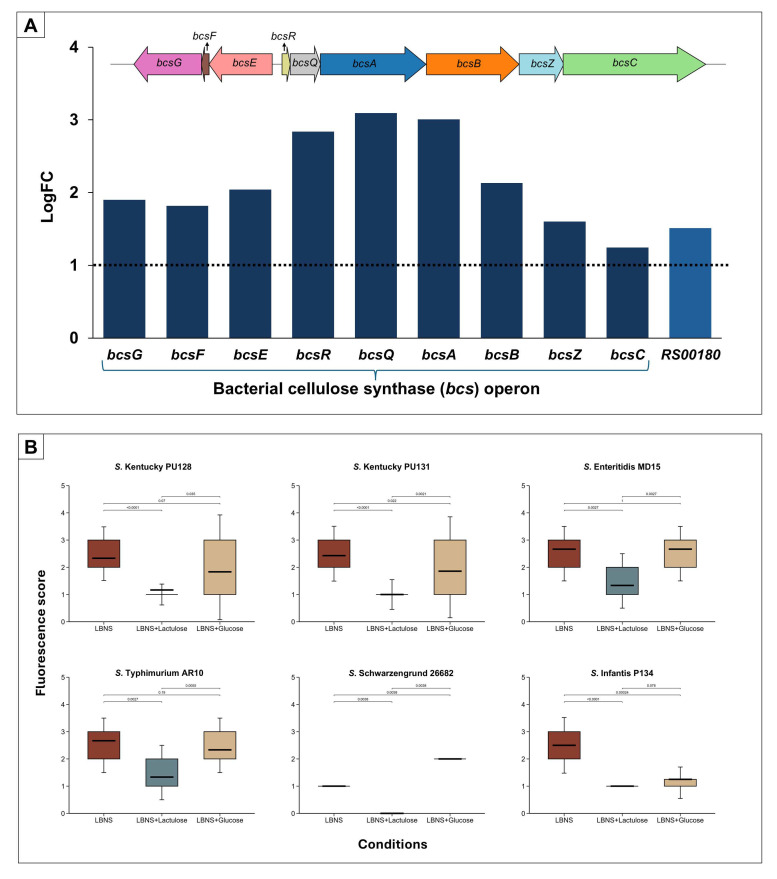
Lactulose exposure induces both cellulose biosynthesis and degradation pathways in non-typhoidal *Salmonella* (NTS). (**A**) Differential expression analysis showing induction of genes within the *bcs* operon, which mediates cellulose biosynthesis, alongside upregulation of *RS00180*, encoding cellulase A, following lactulose exposure in *S.* Kentucky. Bars represent log_2_ fold-change values for individual genes under lactulose-treated conditions relative to control. The dotted line indicates the log_2_ fold-change threshold (log_2_FC = 1). (**B**) Quantification of cellulose-associated fluorescence using a calcofluor binding assay across six NTS serotypes grown at 25 °C on LBNS agar supplemented with calcofluor under three conditions: LBNS alone, LBNS + glucose (20 mM), and LBNS + lactulose (20 mM). Box plots represent fluorescence intensity measurements, reflecting relative cellulose production under each condition.

Besides curli, the NTS biofilm matrix is also composed of cellulose, the expression of which is regulated by the *bcs* operon [103]. Biofilm-associated cellulose in NTS can be readily assessed by Calcofluor white assay, where the dye binds to the β-linked polysaccharides, such as cellulose, and emits fluorescence under ultraviolet light proportional to cellulose abundance [25,103]. In the current study, lactulose induced significant upregulation of the *bcs* operon (Figure 5A), suggesting activation of cellulose synthesis machinery. Concurrently, however, lactulose also induced upregulation of the cellulase-encoding gene (*RS00180*). Given that transcriptional experiments were conducted at 37 °C, the observed concomitant upregulation of the *bcs* operon and *RS00180* is unlikely to reflect biofilm-permissive growth conditions and instead indicates lactulose-driven regulation and suggests that lactulose may promote cellulose remodeling/degradation despite transcriptional induction of synthesis machinery.

We tested this hypothesis by semi-quantitative assessment of cellulose production using the calcofluor assay. The results show that irrespective of the NTS serotype, lactulose supplementation consistently resulted in significantly reduced fluorescence under biofilm-inducing conditions (LBNS, 25 °C) (Figure 5B), indicating a net decrease in cellulose accumulation. This apparent disconnect suggests that lactulose promotes a dynamic state of cellulose turnover rather than stable matrix formation.

Notably, the biofilm master regulator *csgD*, which is known to control both curli and cellulose biosynthesis, was downregulated under lactulose conditions. This suggests that canonical *csgD*-dependent activation of the *bcs* operon is suppressed. The observed upregulation of *bcs* genes may therefore reflect compensatory activation through alternative pathways, such as the diguanylate cyclase *dgcQ* (*yedQ*) (Supplementary File S1) [103,104]. However, the concurrent induction of cellulase activity likely counteracts cellulose synthesis, resulting in reduced net accumulation of extracellular polysaccharides.

Furthermore, downregulation of *csgD* supports a broader suppression of extracellular matrix production, as it coordinates both curli and cellulose biosynthetic pathways. Together with the observed increase in cellulose degradation, these findings indicate that lactulose promotes destabilization of the biofilm matrix through combined suppression of matrix production and enhanced polysaccharide turnover. Additionally, increased expression of cellulose-associated genes under these conditions may reflect a stress-responsive state rather than productive biofilm formation [105]. Collectively, suppression of curli production and increased cellulose degradation are consistent with a model in which lactulose may shift the stress regulon toward net biofilm suppression rather than stable matrix accumulation.

### 3.5. Lactulose Represses Type-1 Fimbrial Gene Expression

**Figure 6:**
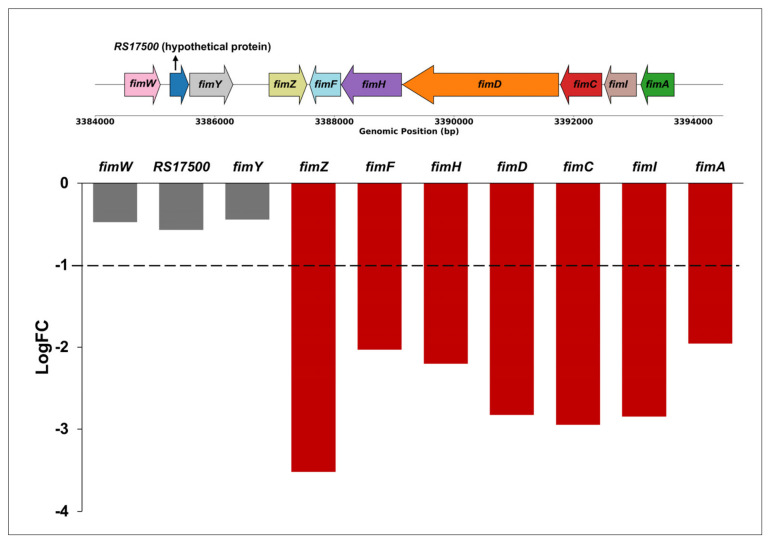
Lactulose exposure repressed the expression of genes encoded on the *fim* operon. The dotted line indicates the differential expression threshold (log_2_FC = −1). The schematic above the plot illustrates the genomic organization of the *fim* operon.

Besides biofilm-associated genes, lactulose exposure resulted in significant downregulation of the *fim* operon, which encodes type 1 fimbriae required for epithelial attachment and invasion in NTS [106]. The *fim* operon comprises ten genes (*fimA*, *fimI*, *fimC*, *fimD*, *fimH*, *fimF*, *fimZ*, *fimY*, *fimW*, and *C1D15_RS17500*) involved in type 1 fimbrial assembly and control [107,108], with the *fimAICDHFZ* transcriptional unit [109] showing marked repression in response to lactulose (Figure 6 and Supplementary File S1). While fimbrial expression is known to respond to environmental factors such as pH and osmolarity [110], culture conditions in this study remained near neutral pH (pH = 6.94–6.98), suggesting that lactulose-induced osmotic pressure may contribute as a driver, although additional regulatory factors cannot be excluded.

Consistent with this, lactulose exposure also led to significant downregulation of *lrp* (Supplementary File S1), a global, nutrient-responsive regulator that links metabolic state to virulence gene expression [111]. Lrp functions as a bimodal transcriptional regulator integrating metabolic signals to control diverse adaptive programs, including those governing fimbrial expression. In NTS, Lrp acts upstream in the regulatory hierarchy controlling type 1 fimbriae by contributing to activation of regulators such as FimZ, which drive *fim* operon transcription [112]. Thus, reduced Lrp levels can be expected to disrupt activation at the *fimZ* promoter, resulting in coordinated shutdown of fimbrial gene expression. Together, these findings support a model in which lactulose likely acts as a metabolic signal that shifts NTS away from fimbriae-mediated host-adherent behavior toward a metabolic-focused state via Lrp-dependent regulation, with reduced Lrp levels indirectly suppressing fimbrial expression through loss of FimZ-mediated activation. Intracellular GlcNAc-6P is also known to suppress activation of the *nagC* gene, which can, in turn, suppress the expression of type I fimbriae [102,113]. Although *nagC* expression was not differentially regulated in this study (Supplementary File S1), this does not exclude involvement of the GlcNAc metabolic pathway, as NagC activity is primarily regulated post-translationally through intracellular GlcNAc-6P rather than changes in transcript abundance [113].

### 3.6. Lactulose Suppresses Flagella-Dependent Motility

**Figure 7:**
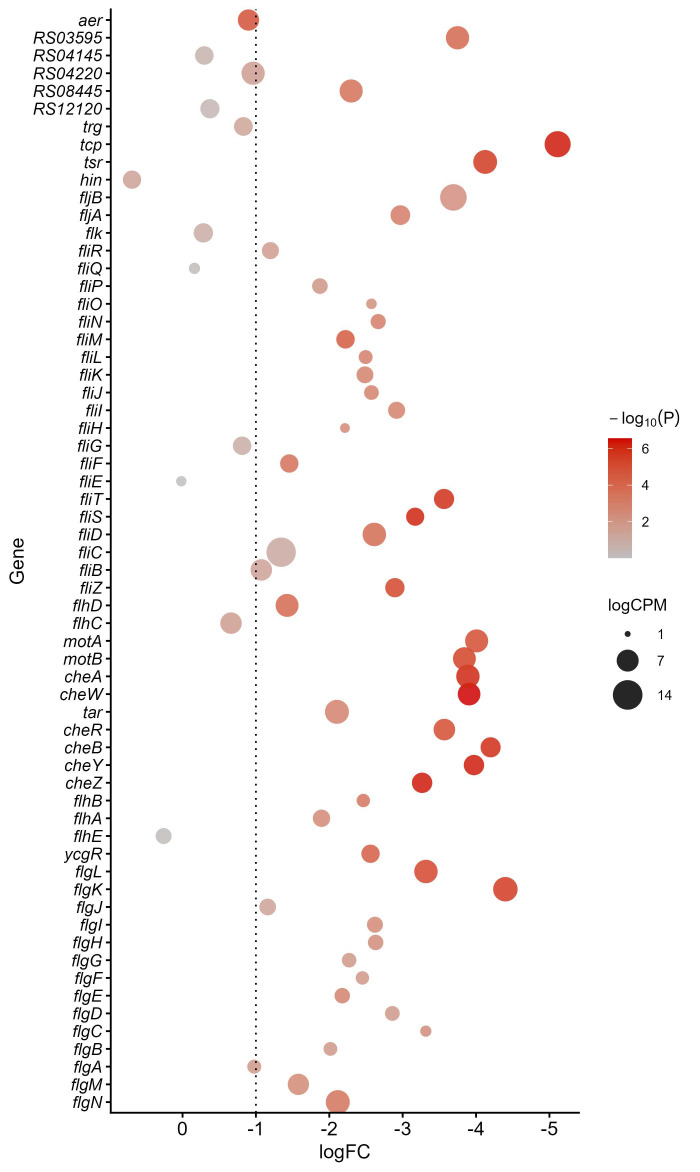
Lactulose exposure represses expression of motility-associated genes. The x-axis on this bubble plot shows log_2_ fold change in expression, and the y-axis shows motility-associated genes. The dotted vertical line indicates the log_2_ fold-change threshold (log_2_FC = 1). Bubble size corresponds to log counts per million (logCPM), reflecting relative transcript abundance, and color intensity (dark red) indicates statistical significance expressed as −log_10_(*p* value).

**Figure 8:**
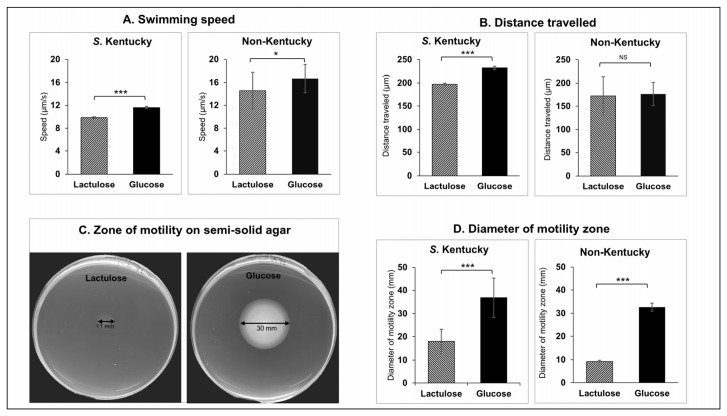
Lactulose exposure suppresses motility in non-typhoidal *Salmonella* (NTS). (**A**) The mean swimming speed of seven *S.* Kentucky strains and four other NTS strains grown in M9 broth supplemented with 20 mM lactulose was significantly reduced when compared to glucose-treated controls (*p* < 0.05). (**B**) The mean total distance traveled by seven *S.* Kentucky strains grown in M9 broth supplemented with 20 mM lactulose was significantly reduced when compared with glucose-treated cultures; this difference was not significant among other NTS strains. (**C**) The motility zone diameter for a representative *S.* Kentucky strain P72 on semi-solid agar plates supplemented with lactulose (11 mm) was significantly smaller when compared to glucose (30 mm). (**D**) The mean motility zone diameter across seven *S.* Kentucky strains and four other NTS strains grown on semi-solid agar plates supplemented with lactulose was significantly smaller when compared with glucose, indicating lactulose-induced motility impairment. The asterisk * sign indicates *p*-value < 0.05, *** indicates *p*-value < 0.001, and NS indicates statistically not significant.

In this study, lactulose exposure induced significant downregulation of the majority of motility-related genes, including components of the *flg*, *flh*, *fli*, *che*, and *mot* operons (Figure 7). At the regulatory level, lactulose downregulated key motility regulators, including *flhDC* and structural genes such as *fliC* [114,115,116]. Based on these data, we tested whether lactulose-induced suppression would affect motility. Lactulose supplementation resulted in significantly reduced swim zone diameters on soft agar (*p* < 0.01) and decreased swimming speed and travel distance in microscopy-based tracking (Figure 8A–D and the Supplementary Video S1). These effects were observed across multiple *S.* Kentucky strains and other NTS serovars. Because flagellar biosynthesis is energetically expensive and also contributes to early surface attachment, repression of the flagellar regulon can simultaneously reduce motility and also limit early steps in biofilm establishment [117]. Both motility and biofilm formation contribute to NTS persistence; thus, lactulose-mediated suppression of these phenotypes raises the possibility that this mechanism may operate *in vivo* and warrants direct validation in appropriate models.

### 3.7. Lactulose Induces Expression of Stress-Responsive Pathways in NTS

In addition to suppressing persistence-associated traits such as biofilm, fimbriae, and motility, lactulose strongly activated stress-responsive pathways. At least 21 of 28 known stress-responsive genes were upregulated (Supplementary File S1), including *nlpE*, *pspB*, *pspC*, *pspG*, and *cpxP*, which are linked to cell envelope stress responses [118,119,120,121]. Lactulose also increased expression of *soxRS*, indicating activation of oxidative stress defenses [122]. Although *soxRS*-dependent post-transcriptional repression of *ompF* has been reported in *E. coli* [123], the concurrent downregulation of *ompF* and upregulation of *soxRS* observed here suggests transcriptional regulation. Together, these findings indicate that lactulose metabolism is accompanied by envelope and oxidative stress adaptation, coinciding with repression of biofilm, motility, and fimbrial expression.

### 3.8. Integrated Mechanistic Framework for Lactulose-Induced Reprogramming of Stress-Responsive Pathways Coupled with Repression of Biofilm, Motility, and Fimbrial Expression

**Figure 9:**
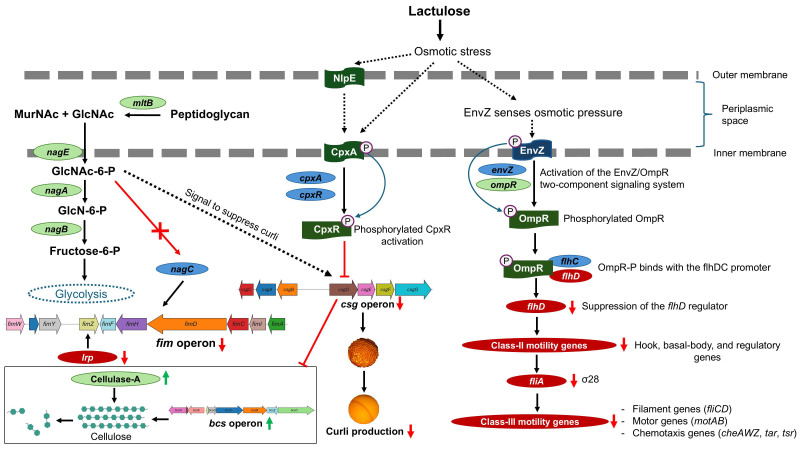
Integrated regulatory model of lactulose-induced osmotic and metabolic stress responses and coordinated repression of motility, curli-dependent biofilm formation, and fimbrial expression in NTS. Lactulose exposure induces osmotic and envelope stress, triggering the activation of the EnvZ/OmpR and Cpx envelope stress response systems. EnvZ/OmpR signaling promotes repression of the flagellar master regulator *flhDC*, resulting in downstream inhibition of flagellar gene expression and reduced motility. In parallel, activation of the Cpx pathway through NlpE, an OmpA-associated outer membrane sensor of the Cpx envelope stress response,, together with metabolic signaling associated with amino sugar flux (including GlcNAc and GlcNAc-6-P), contributes to repression of curli biosynthesis through inhibition of the CsgD regulon. Concurrently, lactulose induced the metabolic repression activity of the leucine-responsive regulatory protein (Lrp), which is predicted to decrease *fimZ* expression, thereby reducing activation of the *fimA* promoter and suppressing type 1 fimbrial production. The box on the bottom left shows upregulation of the *bcs*-mediated cellulose biosynthesis pathway together with increased cellulase A expression, suggesting simultaneous cellulose production and degradation despite downregulation of *csgD*, a known regulator associated with *csg* expression. Collectively, these interconnected regulatory pathways integrate environmental osmotic stress and metabolic state to coordinately suppress motility, extracellular matrix formation, and fimbrial-mediated adhesion while maintaining active carbohydrate utilization. The red arrows indicate downregulated genes/operons, green arrows indicate upregulated genes/operons, and T-bars indicate predicted blocked pathways. Part of the figure was created using Biorender.com.

Collectively, the lactulose-induced transcriptomic landscape (Figure 9) reveals a coordinated system-level reprogramming (Figure 9). At the metabolic level, lactulose exposure activates a broad carbohydrate acquisition and catabolic network (Figure 2), enabling efficient assimilation of lactulose-derived monosaccharides into central metabolism. This metabolic influx is accompanied by signatures of osmotic and envelope stress (Figure 9), including modulation of outer membrane porins (*ompC* upregulation and *ompF* downregulation), consistent with EnvZ/OmpR-mediated adaptation to altered extracellular solute conditions [124]. Concurrent induction of trehalose biosynthesis further supports activation of osmoprotective responses, indicating a shift toward stress-adapted metabolic processing (Figure 2).

This metabolic state is tightly coupled with suppression of motility (Figure 9). Downregulation of *flhDC* and downstream flagellar genes indicates repression of the flagellar regulon, consistent with integration of osmotic and envelope stress signaling into motility control circuits [124,125]. Functionally, this is reflected in the observed reduction in swimming capacity (Figure 8), supporting a shift away from energy-intensive motility toward nutrient assimilation. In parallel, lactulose exposure suppresses key surface colonization systems, including expression of curli and type-1 fimbrae (Figure 9). Strong downregulation of the *csg* operon indicates inhibition of curli biosynthesis via repression of the *csgD* regulatory axis, a central hub controlling extracellular matrix formation [95,96]. This is likely linked to metabolic rerouting of amino sugar flux, including altered GlcNAc-6-phosphate signaling through the *nag* pathway, linking cell wall recycling to biofilm regulation [102,126]. Envelope stress pathways, including Cpx system activation, further support the repression of both curli and motility regulon through downstream inhibition of *flhDC* and *csgD* [127,128,129]. On the other hand, suppression of type-1 fimbrial expression is likely through downregulation of global regulators such as Lrp, which influences activation of the FimZ–FimA regulon [111,112]. This suggests that lactulose-driven metabolic signals extend beyond biofilm and motility regulation to broadly attenuate host-adaptive adhesion mechanisms.

Importantly, these regulatory shifts occur despite continued carbon utilization, indicating a decoupling of metabolic activity from persistence-associated phenotypes. Rather than reflecting growth limitation, lactulose exposure likely drives a reallocation of cellular resources toward carbohydrate metabolism and stress adaptation at the expense of motility, biofilm formation, and adhesion. Together, these data are consistent with a unified model in which lactulose functions as both a metabolizable substrate and a regulatory cue that reprograms NTS physiology toward a metabolically active state with reduced expression of biofilm-, motility-, and fimbrial-associated programs rather than a fully established non-persistent state (Figure 9).

## 4. Conclusions

This study provides a transcriptome-wide characterization of lactulose-responsive gene expression in NTS, identifying candidate transport systems and carbohydrate-active enzymes potentially involved in lactulose uptake and catabolism. These findings are consistent with a multi-component carbohydrate utilization network enabling NTS to metabolize lactulose as an alternative carbon source. Beyond metabolism, lactulose induces broad transcriptional reprogramming associated with the suppression of traits associated with persistence and host colonization under in vitro conditions. This includes coordinated repression of *csgD*-dependent curli biosynthesis and the associated extracellular matrix, alongside reduced flagella-mediated motility and type 1 fimbrial expression through downregulation of the *flhDC* regulon and fimbrial regulatory networks, respectively. Extracellular matrix remodeling also extends to cellulose, where transcriptional induction of the *bcs* operon is accompanied by increased cellulose-degrading activity and reduced calcofluor fluorescence, indicating disruption of cellulose-dependent matrix stability and impaired matrix assembly.

Collectively, lactulose supports metabolic activity while suppressing the integrated extracellular matrix system composed of curli and cellulose, as well as motility and fimbrial programs. This is consistent with a partial decoupling of carbon utilization from persistence-associated phenotypes, resulting in a metabolically active state with reduced expression of biofilm-, motility-, and fimbrial-associated programs rather than a fully established non-persistent state. Overall, this work proposes a mechanistic framework based on transcriptomic inference and provides a foundation for future functional validation and in vivo studies.

## Figures and Tables

**Table 1:** Wild-type non-typhoidal *Salmonella* (NTS) strains used in this study.

Serotype	ST	Strain_ID	Study Purpose	Reference
*S.* Kentucky	ST198	PU61	RNA-seq and motility	[23]
*S.* Kentucky	ST198	PU128	RNA-seq, motility, and biofilm	[23]
*S.* Kentucky	ST198	PU131	RNA-seq, motility, and biofilm	[23]
*S.* Kentucky	ST152	P55	RNA-seq and motility	[23]
*S.* Kentucky	ST152	P72	RNA-seq and motility	[23]
*S.* Kentucky	ST152	PU116	RNA-seq and motility	[23]
*S.* Kentucky	ST152	PU363	RNA-seq and motility	[23]
*S.* Enteritidis	N/A	MD15	Biofilm and motility	[23]
*S.* Typhimurium	N/A	AR10	Biofilm and motility	[25]
*S.* Schwarzengrund	N/A	26682	Biofilm and motility	[23]
*S.* Infantis	N/A	P134	Biofilm and motility	[25]

## Data Availability

The original contributions presented in this study are included in the article and as Supplementary Files. Further inquiries can be directed to the corresponding author: Devendra H. Shah (devendra.shah@ttu.edu).

## Supplementary Materials

The following supporting information can be downloaded at: https://www.mdpi.com/article/10.3390/pathogens15060592/s1.
